# In the heat of the moment: Including realistic thermal fluctuations results in dramatically altered key population parameters

**DOI:** 10.1002/ece3.70124

**Published:** 2024-08-27

**Authors:** Sam P. Boerlijst, Eline Boelee, Peter M. van Bodegom, Maarten Schrama

**Affiliations:** ^1^ Department of Environmental Biology, Center for Environmental Research Leiden University of Leiden Leiden the Netherlands; ^2^ Division of Inland Water Systems Deltares Delft the Netherlands

**Keywords:** Arduino, *Culex pipiens*, heat wave, larval development, mesocosm, microcontroller, mosquito, temperature fluctuations

## Abstract

Temperature is commonly acknowledged as one of the primary forces driving ectotherm vector populations, most notably by influencing metabolic rates and survival. Although numerous experiments have shown this for a wide variety of organisms, the vast majority has been conducted at constant temperatures and changes therein, while temperature is far from constant in nature, and includes seasonal and diurnal cycles. As fluctuating temperatures have been described to affect metabolic processes at (sub)cellular level, this calls for studies evaluating the relative importance of temperature fluctuations and the changes therein. To gain insight in the effects of temperature fluctuations on ectotherm development, survival, and sex ratio, we developed an inexpensive, easily reproducible, and open‐source, Arduino‐based temperature control system, which emulates natural sinusoidal fluctuations around the average temperature. We used this novel setup to compare the effects of constant (mean) temperatures, most commonly used in experiments, block schemes, and natural sinusoidal fluctuations as well as an extreme variant with twice its amplitude using the cosmopolitan mosquito species *Culex pipiens* s.l. as a study organism. Our system accurately replicated the preprogrammed temperature treatments under outdoor conditions, even more accurately than traditional methods. While no effects were detected on survival and sex ratio within the ranges of variation evaluated, development was sped up considerably by including temperature fluctuations, especially during pupation, where development under constant temperatures took almost a week (30%) longer than under natural fluctuations. Doubling the amplitude further decreased development time by 1.5 days. These results highlight the importance of including (natural) oscillations in experiments on ectotherm organisms – both aquatic and terrestrial – that use temperature as a variable. Ultimately, these results have major repercussions for downstream effects at larger scales that may be studied with applications such as ecological niche models, disease risk models, and assessing ecosystem services that rely on ectotherm organisms.

## Graphical abstract

Emulating natural fluctuations demonstrated accelerated development in *Culex pipiens* s.l. mosquitoes, underscoring the necessity of incorporating natural oscillations in studies of ectotherm organisms for more accurate ecological and disease risk assessments.

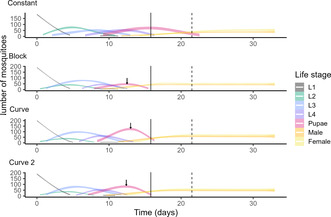

## 1 INTRODUCTION

Temperature is commonly acknowledged as one of the primary forces driving ectotherm populations (Mellanby & Gardiner, 1997; Newell, 1966). Temperature influences metabolic rates at cellular and subsequently organismic level (Kuznetsov et al., 2016) as a result of differences in optimal temperatures for different enzymatic reactions (Rao & Bullock, 1954). In extreme cases, survival might be affected, as a result of denaturation and impaired membrane function (Bowler, 2018). Experiments using temperature are of major importance to predict the effects of large‐scale disturbances like climate change on these organisms.

Global warming affects not only daily mean temperature but also the daily interval in temperature (Colinet et al., 2015; Easterling et al., 1997; Paaijmans et al., 2013). Additionally, effects on a local scale exist, wherein effects like urban heat islands affect diurnal and seasonal temperature fluctuations. Various studies suggest that indeed the diurnal amplitude also might affect the development of ectotherm organisms (Kern et al., 2015; Kingsolver et al., 2015; Kuznetsov et al., 2016; Ludwig & Cable, 1933; Waqas et al., 2020), possibly via temperature‐dependent processes such as growth and cell differentiation (Ratte, 1984; van der Have & de Jong, 1996). Similar, and possibly sex‐specific effects (Agnew et al., 2000; Alcalay et al., 2018), have been detected for mosquito vectors (Colinet et al., 2015; de Majo et al., 2019; Headlee, 1941; Huffaker, 1944; Ratte, 1984), which undergo their subadult development in shallow (often temporary) water bodies where drastic temperature changes are common. To understand how to manage such anthropogenic impacts, it is thus crucial to understand the exact effects of temperature fluctuations on arthropod vector development. However, a small‐scale and inexpensive experimental system to do so was until recently unavailable (Hagstrum & Hagstrum, 1970; Hermann et al., 2022).

Most ecological experimental studies including temperature as a variable (i.e., micro‐ and mesocosms) have hitherto been dependent on decentralized temperature regimes in climate cabinets (Greenspan et al., 2016; Hagstrum & Hagstrum, 1970) or with heating elements set to a constant temperature (Bayoh & Lindsay, 2004; Brust & Kalpage, 1967; Impoinvil et al., 2007; Shapiro et al., 2017; Shelton, 1973). The latter system occasionally has been adapted to a block scheme, where temperatures fluctuate between two levels that are fixed over a set amount of time (Alcalay et al., 2018; Spanoudis et al., 2019) or by physically moving the study organism between climate chambers (Niederegger et al., 2010). However, the widespread availability of micro‐controllers (Bolanakis, 2019) allows for a well‐replicated assessment of the relative importance of thermal variation.

To better understand the precise effects of temperature on ectotherm development, we developed an inexpensive, easily reproducible, and open‐source Arduino‐based temperature control system. This setup allows for emulation of natural sinusoidal fluctuations above ambient temperatures while keeping the number of degree‐days over all treatments the same. Here, as a case study to validate the metabolic effects, we compared the effects of commonly used constant (mean) temperature and block schemes with natural sinusoidal fluctuations, as well as an extreme regime with twice its amplitude. We used the mosquito species *Culex pipiens (hereafter Cx. pipiens)*, a cosmopolitan vector for a range of viral pathogens including West Nile virus, Sindbis virus, and Usutu, as a model species. As the subadult stages of this species are aquatic, this allows for easy implementation of temperature regimes via immersible heating elements.

## 2 MATERIALS AND METHODS

To study the effects of natural temperature oscillations on metabolic rates in aquatic systems, we used the following procedure.

### 2.1 Temperature control system

Our novel temperature control system (holistic intermittent heatwave instrument; hereafter HIHI) for investigating the effects of temperature fluctuations is comprised of a closed container to hold the electronics, a power supply, the internal electronics, and relay‐controlled power strips. The setup allows for up to eight groups of heaters to be connected per HIHI, for a total of 10 Ampère per group. Using 200 W heaters, this translates to 80 mesocosms in total.

#### 2.1.1 Container

As the HIHI may be used outdoors, care was taken to protect the electronic components and their connections from rain/humidity while preventing build‐up of heat. Two polycarbonate storage boxes were used to house the electronic components, one for the microcontroller and relay board, and one for the power strips and their connection to the heaters. Holes were cut into the bottom of both boxes for ventilation, and into the overhang of the lid to allow wires to pass through. The cables were glued in place with hot glue to prevent moisture from entering. To allow for sufficient air supply, holes were cut on the bottom of the containers and were placed on a layer of stones to allow for aeration. Placement of the containers was limited by the length of the power cables and cables of the temperature sensors. In our case, we placed the containers in the middle of the experimental setup.

#### 2.1.2 Power supply

HIHI is operated by one 5v micro‐USB power supply, which may be connected to a laptop to allow for logging the temperatures from the serial logger included in the Arduino IDE or may be connected to a 1A phone charger. The relay board operates on grid power (240 V).

#### 2.1.3 Control box

**FIGURE 1:**
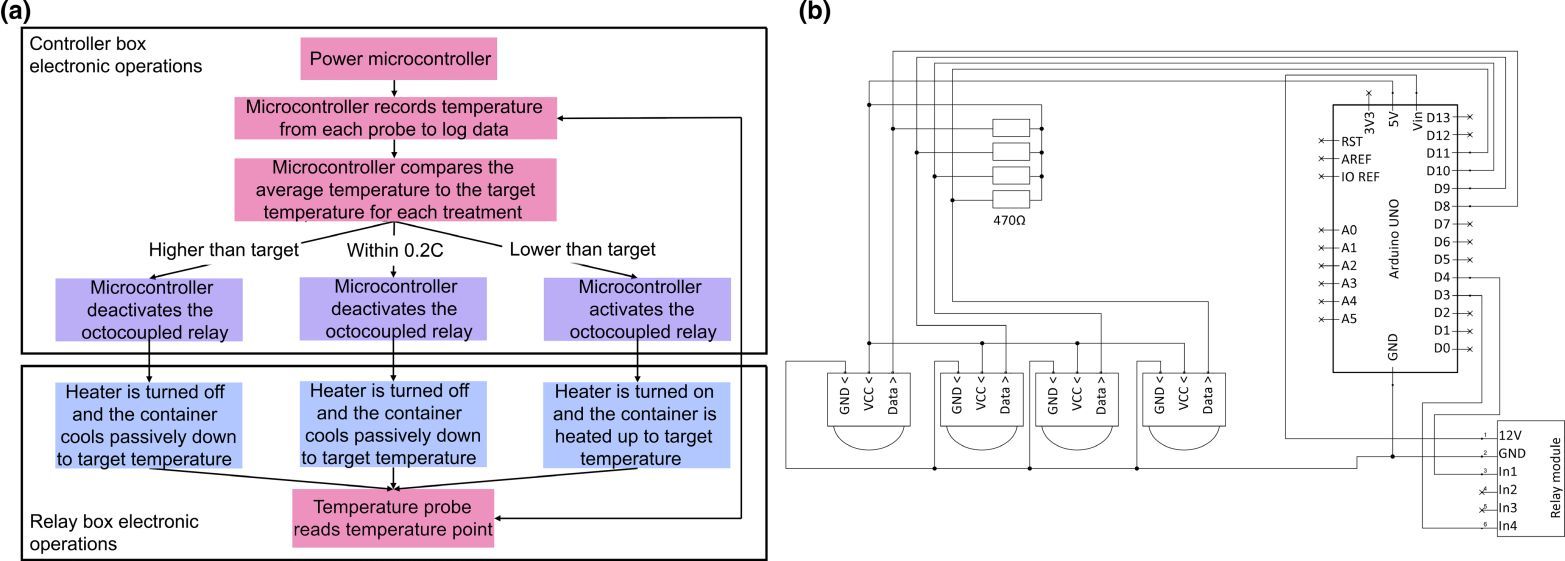
Schematic overview of (a) the operational process of the temperature controller and (b) the internal electronics and their connections.

The control box internal electronics are shown in Table 1 and Figure 1. The costs for the electronics are estimated at 49 euro. The programmed temperature is compared to the current surface water temperature, measured by DS18B20 (i‐Button) sensors (Maxim Integrated). Based on this information, each treatment is heated or left to (passively) cool via activation or deactivation of the heaters connected via an optocoupled relay with a specified interval.

**TABLE 1:** Components for the temperature controller.

Component	Quantity	Price (€)	Specifications	Use
Arduino uno	1	24.95		Controls temperature
Temperature sensor	4	4 × 2.95	DS18B20; minimum of two per treatment	Measures temperature
Optocoupler relay module	1	10.00	2ph109375a or equivalent; 240 V/10A per relay; 2 channel or more	Turns heaters on or off
Resistors	4	4 × 0.03	470 Ω	Limits current to sensors
perfboard	1	0.70	Size approximately 4 × 6 cm	To assemble circuit onto
Insulated conductor cable	1	0.60	1 m	Connects components
Female header pin	1	0.18	1 × 6 pins	Connector to relay module
	Total price	48.35		

#### 2.1.4 Relay‐controlled power strips

The power strips are driven by opening the live wire and connecting these to the COM and NO connections of their respective relay group. Heaters were plugged into the power strips. Care was taken to adequately distribute the heaters over multiple relays as not to overload the relay and/or power strip (commonly rated for 10A and 16A, respectively).

#### 2.1.5 Operational process

The operational procedure that the temperature controller undergoes is specified in Figure 1a. The microcontroller can be pre‐programmed with user‐specified temperature schemes for each time interval (e.g., 1, 2, or 5 min). The operational code needed to operate the temperature controller may be downloaded from GitHub and uploaded using the open‐source Arduino integrated development environment (Wheat, 2011; see data accessibility section). Temperature schemes can be altered to any temperature scheme, for instance real‐world thermal regimes captured from environmental data loggers, by changing the temperature arrays. Diurnal temperature fluctuations can be programmed with the “curve calculation” Excel file provided in the dryad repository, by changing the interval, daylength, mean temperature, and amplitude and subsequently copying the produced array to the code.

### 2.2 Validation tests

Two exploratory studies were performed as generic validation to (1) determine the interval with which the temperature controller should operate and (2) to determine and correct for any bias present in the programmed temperatures. These tests were performed in white polypropylene carbonate 12 L buckets (31.6 × 32.5 × 25.5 cm), which were also used for the proof of principle experiment.

#### 2.2.1 Interval calculation

The interval for the temperature controller was determined by recording the time needed to either warm or passively cool the 12 L bucket by 0.1°C. This was done by taking the average time over 2°C heating/cooling. Using 200 W HS‐aqua heaters, a 2‐min interval proved sufficient, as this allowed the container to either cool by 0.1°C or to heat up by 0.08°C during the allotted time. From this, a heat transfer coefficient could be derived to predict intervals that may be used for similar setups (surface, volume, and humidity) under a range of temperatures as compared to ambient air temperature (Table 2).

**TABLE 2:** Predicted intervals to cool 0.1°C derived from Newton's law of cooling, a heat transfer coefficient of 623,587 W/(m^2^K), a diameter of 32 cm, relative humidity of 68%, and variable temperature interval as compared to ambient air temperature.

Temperature difference (°C)	Interval (mins)
20	0.69
18	0.77
16	0.87
14	1.00
12	1.16
10	1.40
8	1.75
6	2.34
4	3.52
2	7.13

#### 2.2.2 Bias correction

Adherence to the programmed temperatures specified in section 2.3 was validated by use of a one‐day pilot. The setup was allowed to run normally, and the number of degree‐days, equal to the sum of the mean temperature per 15 min for a 24‐h period, was estimated for each treatment by calculating the approximation of the surface between each (2‐min interval) timepoint as a trapezoid. Using this data, a bias of +0.1C was found, after which the formulas were corrected, and the pilot was run again to validate that the bias had been reduced (Table S1).

### 2.3 Temperature treatments

**FIGURE 2:**
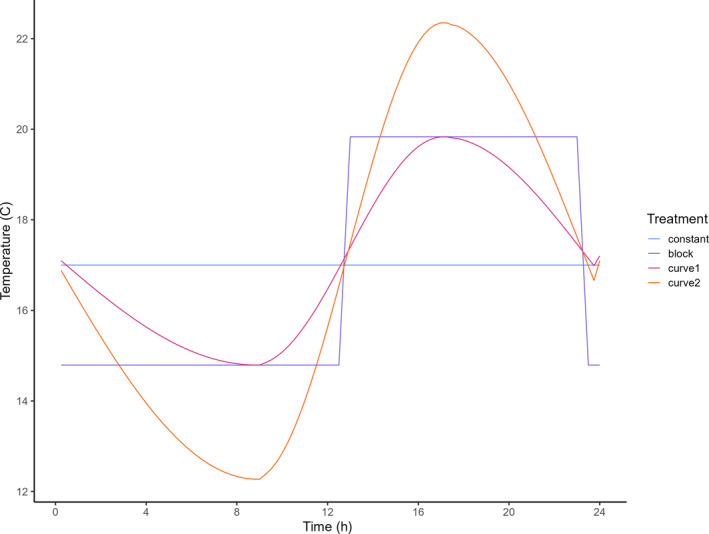
Visual representation of the programmed temperature regimes of increasing levels of temperature fluctuation. The area under the graph – indicative for the amount of energy in the aquatic system – is the same for all treatments. The temperatures mimic an average day in June (2020) in the Netherlands.

Recent insect development models propose that ectotherm metabolism and development do not respond additively to temperature fluctuations (Wu et al., 2015), and are dependent on a variety of temperatures (Kuznetsov et al., 2016; Ludwig & Cable, 1933; Newell, 1966; Waqas et al., 2020; Wu et al., 2015) for optimal cell growth and differentiation (Ratte, 1984; van der Have & de Jong, 1996). This could result in variation across populations as a result of (local) adaptations (Sternberg & Thomas, 2014). As to our knowledge, an optimum diurnal temperature fluctuation has hitherto not been established for our model species, we chose to simulate as much realism as possible and thus used temperatures associated with the peak of the mosquito season for our latitude. Because of this, we simulate an average day at the peak of the Dutch mosquito season. To ensure the number of degree days to be consistent across the four treatments of increasing fluctuation, we used the following procedure. Based on aquatic surface temperatures measured in May and July 2020, we determined mean, minimum, maximum temperature, and the temporal interval between these (Figure S1). These temperature metrics were used to create four treatments with equal mean (Figure 2) using the methods described in the operational code (see data availability section).

### 2.4 Proof of principle experiment

**FIGURE 3:**
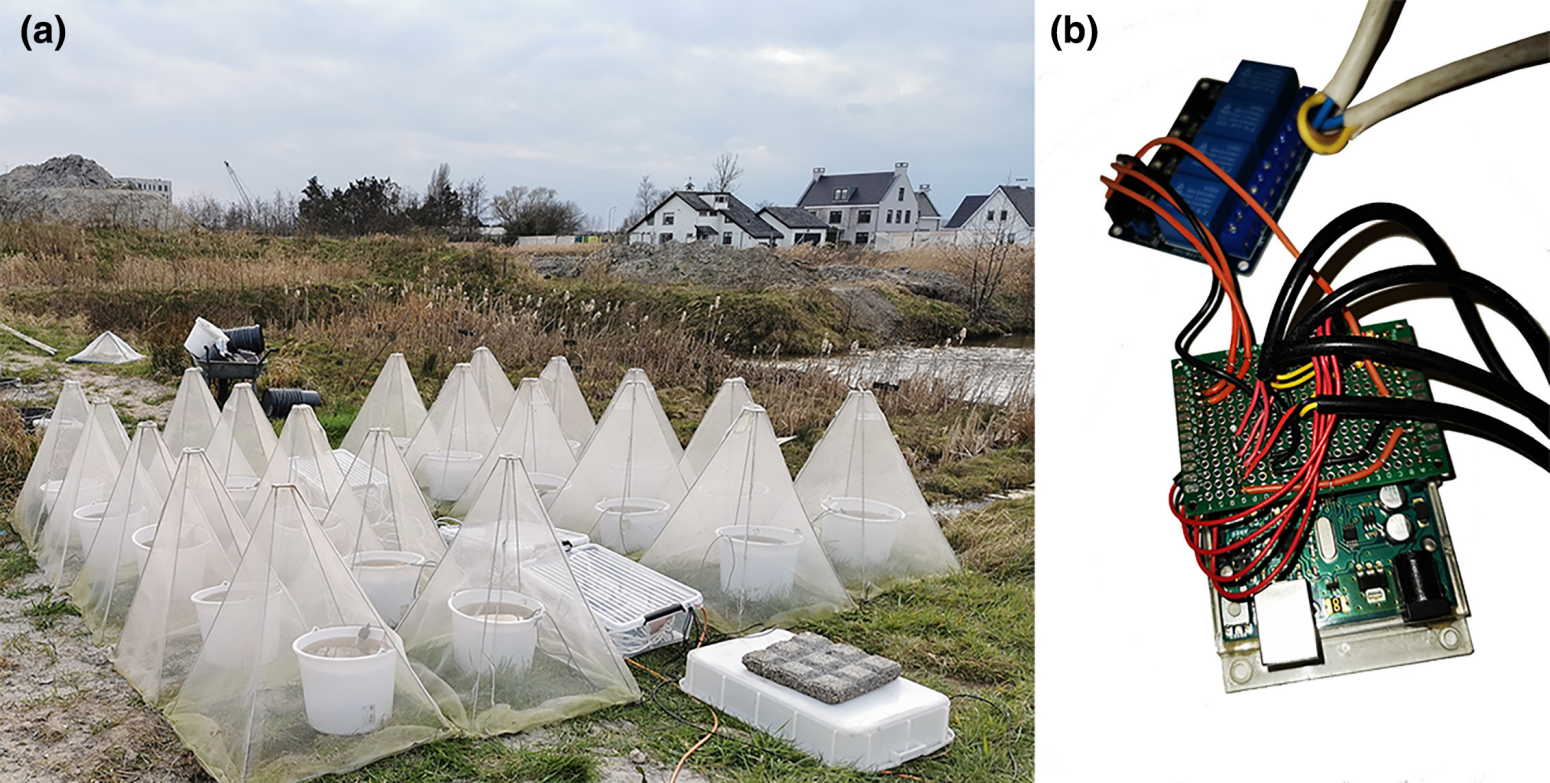
Overview of the experimental setup. Panel (a) shows the mesocosms covered by emergence traps and the HIHI and power strips in the middle. Panel (b) shows the HIHI, with the Arduino and circuit board to the left, and the relay board to the right.

In order to validate the reliability of the proposed system, and the effect of temperature fluctuations on the development and (sex‐specific) survival of an ectotherm organism, a proof‐of‐principle experiment was conducted at the living lab field station of Leiden University, the Netherlands (Boerlijst et al., 2023). The current experiment used the mosquito *Cx. pipiens* as a model organism. The experiment took place between the end of March and beginning of May 2021 and took 34 days. As the outside temperatures are relatively low in that period, this allowed us to mimic the natural temperature conditions of an average day in June 2020, because the ambient temperatures are sufficiently low to cool down the buckets to the desired temperatures. June is commonly regarded as the optimal month for mosquito development in NW Europe as the amount of sunlight energy, a direct determinant of the water temperature, is at its maximum (Becker et al., 2010). The experiment, containing four temperature treatments (Figure 2), had five replicates each consisting of white polypropylene carbonate 12 L buckets (31.6 × 32.5 × 25.5 cm; Figure 3 and Figure S2). The containers are representative of the artificial containers that *Cx. pipiens* is known to colonize (Koenraadt & Harrington, 2008). Within these small, temporary water bodies, fluctuation of temperature is highest and there is little competition and predation (Kumar & Hwang, 2006).

Each bucket was filled with 10 L of dechlorinated tap water and a standardized community of algae and bacteria, collected with a plankton net (250 μm with a 53 μm collector) from a neighboring lake (Dellar et al., 2022). The filtered algae and bacteria obtained were divided equally over all containers so that 1 L of water in the set‐up contained as many microbes as a liter of ditch water. An eutrophic environment (20 mg/L N‐total; Loeb & Verdonschot, 2008) was created, using cow manure pellets (2.4% N; 1.5% P_2_O_5_; 3.1% K_2_O) to minimize intraspecific competition (Boerlijst et al., 2023). The buckets were thereafter covered with a 0.1 mm mesh to prevent natural colonization and left to acclimatize for 1 week. 200 first instar larvae were then added to each container and the four treatments were semi‐randomly assigned within a Latin square design (Figure S2). During the experiment, an emergence trap (Cadmus et al., 2016) was used to prevent colonization, protect the mosquitoes from predators, and prevent the emerged mosquitoes from flying out. Evaporated water was replenished daily using dechlorinated tap water stored at ambient temperature.

Life‐stage‐specific development and sex‐specific emergence rates were included as dependent variables. Additionally, dissolved oxygen concentration, turbidity, and chlorophyll α concentration were measured as indicators for resource competition due to their relation with bacterial and algal metabolism (Ansa‐Asare et al., 2000; Coolidge, 2017).

#### 2.4.1 Study organism

*Culex pipiens* var. *pipiens* egg rafts were obtained from the rearing facility of Wageningen University, the Netherlands. The larvae were subsequently allowed to hatch in a white plastic bucket containing 10 liters of lake water where they were kept at ambient temperature until the start of the experiment.

#### 2.4.2 Measurements

The temperature in each mesocosm was recorded every 15 min for the duration of the experiment by a temperature logger (iButton DS1921G#F5D) placed 5 cm under the water surface to (i) measure the temperature near the water surface where mosquito larvae spend most of their time (Becker et al., 2010) and (ii) prevent the loggers from emerging due to evaporation. Larval development was measured 5 days a week and time to pupation, time to emergence, survival rate, and sex ratio were determined congruent with the methods of Boerlijst et al. (2023) and Dellar et al. (2022). Dissolved oxygen concentration (DOC), chlorophyll α, and turbidity levels were measured weekly with a Hach HD40 and Aquafluor 8000–010, respectively, using manufacturers’ protocols.

### 2.5 Statistical analysis

**FIGURE 4:**
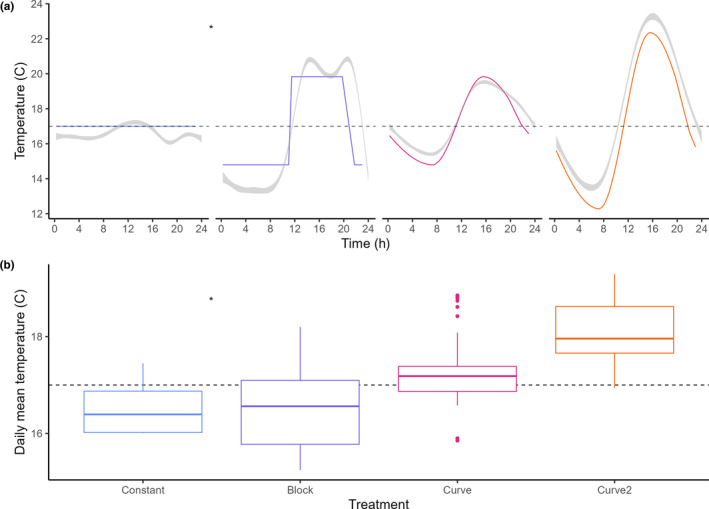
Measured temperatures as compared to the programmed *t* temperature regimes of increasing levels of temperature fluctuation. Panel (a) shows the measured temperatures over time, including standard error, in gray, and the programmed regimes in color. Panel (b) shows the mean daily temperature in color for the temperature sensors shown in gray. *Subset of first 10 days of the experiment (up to the short circuit of the respective treatment.

All data were analyzed in R version 4.04 (R Core Team, 2018). To compare the amount of energy per day between the different treatments, we calculated the degree days in unit of hour per day per mesocosm as approximated integral of the iButton measurements with trapezoidal integration from the pracma package (Borchers, 2022). Adherence of the treatments to their respective programmed temperatures was analyzed using an ANOVA on a linear mixed effect models with the formula: Temperature ~ time + predicted temperature + (1|Cosm) + (1|Day) for the block, curve, and curve 2 treatments. As all predicted values for the constant treatment are equal to its mean, we analyzed this treatment using a Wilcoxon rank sum test. The block treatment was analyzed using the (10) days prior to the short circuit of this treatment (Figure 4a). Effects on the life history of the within‐day variation within the full experiment (Figures S3 and S4) are described in the discussion. Daily mean temperature for each of the respective treatments was assessed using a two‐way ANOVA using the formula Degree days ~ Day + Treatment + Day:Treatment. Data from day eight was excluded as a blown fuse within the field facility had disrupted the block and constant treatments.

Differences in time to pupation, time to emergence, survival, and sex ratio were assessed with general linear models comparing the number of emerged mosquitoes, average development stage, the number of emerged mosquitoes per sex respectively while using DOC, chlorophyll α, and turbidity as main factors. Covariates and their interactions were stepwise removed from the full models during model optimization if not significant and if the Akaike information criterion indicated a worse fit of the data. All full models are presented in Table S2.

## 3 RESULTS

To assess the effect of temperature fluctuations on culicid metabolic rate, data was collected on (1) temperature, to allow for comparison with target temperatures and thus HIHI accuracy and (2) life‐history traits to detect developmental differences across different levels of thermal fluctuation.

### 3.1 Temperature series

To compare the reliability of our proposed system, we compared predicted and actual temperature measurements per 15 min for each treatment (Figure 4a). A 0.5°C difference was detected in temperature between the predicted and actual measurements (*W* = 5963, *p* < .001). No significant differences were detected for the block (*F*_1,1_ = 0.01, *p* = .94), constant (*F*_1,1_ = 0.003, *p* = .97), curve (*F*_1,1_ = 0.43, *p* = .63) and curve 2 (*F*_1,1_ = 1.63, *p* = .42) treatments.

The daily mean temperatures derived from the iButtons were subsequently compared per day across treatments with increasing temperature fluctuation to detect differences in energy input (Figure 4b). Differences were detected over time (*F*_20_ = 8.972; *p* < 2e‐16; power = 1) across treatments (*F*_3_ = 144.66; *p* < 2e‐16; power = 1), also interactively (*F*_60_ = 4.759; *p* < 2e‐16; power = 1). Post hoc pairwise t‐tests indicated differences between Constant and the treatments curve and curve 2 at day 3 and 7 (*p* < .05). Further differences were detected between Block and the treatments Constant from day 11 onwards (*p* < .05), Curve at day 9–18, 20 and 22 (*p* < .05) and curve 2 at day 9 (*p* < .05).

### 3.2 Life‐history effects

Absolute survival rate and sex ratio were not impacted by different levels of temperature fluctuation within the ranges tested (*p* > .1). No differences in DOC, chlorophyll, and turbidity were found between the treatments.

**FIGURE 5:**
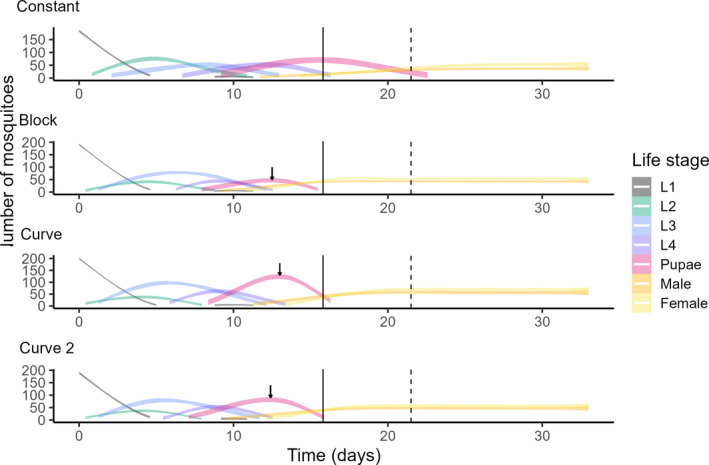
Counts per life stage for constant, block, curve and curve 2. As mortality over time was not measured, the sum of the life stages at each timepoint has been assumed to be equal to the total number of emerged adults for each respective container, except for day 0, which is equal to the starting density. Subsequently, for visualization purposes, all mortality is assumed to occur between day 0 and 1. Median time to pupation and 95% pupation in the control treatment are indicated by solid and dashed lines respectively. Median time to pupation in the other treatments are indicated by a purple arrow.

Increasing levels of temperature fluctuation decreased development time up to pupation (Figure 5). Differences were detected between the constant and curve treatments (χ^2^ = 2.017, *p* = .022) and the constant and curve 2 treatments (χ^2^ = 2.711, Df = 3, *p* = .003). Increasing levels of temperature fluctuations also lowered time to emergence [*F*(3, 14) = 230.7, *p* < .001, partial ω^2^ = 0.833, power = 1]. Post hoc analysis indicated differences between the constant treatment and all other treatments (*p* < .001, Bonferroni correction) and between the block and curve treatments (*p* < .05, Bonferroni correction). Differences in development time exacerbated during pupation (Figure 5, Figures S5 and S6 and Table 3).

**TABLE 3:** Differences in median time to pupation (∆TTP) and median time to emergence (∆TTE) for each contrasting treatment. Increase/decrease indicates whether differences enlarged or reduced during pupation.

Treatment	∆TTP (days)	∆TTE (days)	∆TTE‐∆TTP (days)	∆TTE / ∆TTP (%)	Increase/decrease
Block – Constant	−2.4	−7.3	−4.8	291	Increase
Block – Curve	−0.5	−2.2	−1.7	440	Increase
Block – Curve 2	0.2	−0.6	−0.8	−300	Decrease
Constant – Curve	2.0	5.1	3.1	253	Increase
Constant – Curve 2	2.6	6.7	4.0	247	Increase
Curve – Curve 2	0.7	1.6	0.9	229	Increase

## 4 DISCUSSION

Here we developed a system to assess the effect of thermal fluctuations on ectotherm metabolic rate. Our HIHI system accurately replicated the preprogrammed temperature treatments under outdoor conditions. Comparatively, it performs even more accurately than the traditional Constant and Block regimes, which tend to overshoot and overcompensate due to the thermostat its accuracy of approximately 1°C (Figure 4a) as compared to the 0.5°C accuracy of the temperature sensor in the proposed setup. The new system was successfully applied to assess the impact of temperature fluctuations on the development of *Culex* mosquito larvae.

Fluctuations in temperature in our proof‐of‐principle study had large effects on larval development time, in line with recent models of insect development (Colinet et al., 2015; Kuznetsov et al., 2016; Vajedsamiei et al., 2021; Waqas et al., 2020; Wu et al., 2015), but we did not detect any effect on survival or sex ratio. Median time to pupation decreased with increasing levels of fluctuation from 16 to 13 days. This effect became even larger during pupation itself with median time to emergence ranging from 22 to 14 days. This is in line with the notion of Kingsolver et al. (2015), that the effect of temperature fluctuations varies across developmental stages. Natural (curve) temperature fluctuations resulted in an average decrease of 7 days (or a third of the development time), as compared to constant temperatures. For both time to pupation and time to emergence, most of the differences in development appear when comparing the constant to natural levels of fluctuation. When comparing Constant to Curve 2, thus doubling the amplitude, development decreased by another 1.5 days. Development under the block treatment resembled the natural fluctuation remarkably well, which might partially be explained by the deviation from the preprogrammed mean temperature and occasionally higher amplitude as a result of the short circuit for this treatment after the first 10 days (Figures S3 and S4). However, the effects of the treatment surpass the change in development rate that might be explained by just a 2°C increase alone (Loetti et al., 2011), which suggests that there are additional biological processes at play, with life‐stage‐specific effects.

The remarkable difference during the pupation phase sheds light on the required additional biological explanation. Pupae solely metamorphize, as their pupal enclosure only allows for respiration, not feeding (Becker et al., 2010). This indicates that interaction with their environment is limited during this stage. As such, the difference in development time with different temperature fluctuations during this life stage is largely internally regulated. Although temperature fluctuations have been described to affect metamorphosis disproportionally (Banahene et al., 2018; Niederegger et al., 2010), we hypothesize that larval stages might be more severely affected than currently visible. During the stages prior to metamorphosis, by other interactions with the environment (competition, food availability, etc.), because of which could mask the effect of thermal fluctuations is less noticeable. Below we elaborate on the possible biological processes underlying the effects of temperature fluctuations on metabolic rate.

Plastic responses to thermal stress, as a part of environmentally induced phenotypic change (plasticity), have been previously described in other ectotherm organisms to be correlated to gene activation, sometimes leading to life‐stage‐specific tolerances (Arias et al., 2011). Although some gene‐specific responses and adaptations are documented (Clark & Roger Worland, 2008), there is a very poor understanding of system‐wide responses (Gracey et al., 2004). It might be assumed that combined with enzymatic activity (Rao & Bullock, 1954), adaptations like gene activation and its effect on metabolic rate might make development under (natural) variable temperatures more favorable. The exact modus operandi behind this pattern, and the relative importance of temperature and genetic dependence on temperature fluctuations, however, remains unknown and requires further study.

Overall, our results strongly suggest that including thermal oscillations in experiments likely results in substantial differences in estimations of key life history parameters (e.g., development time), in our case for mosquitoes. Based on a large body of historical (pre‐1970s) as well as more recent literature, there are good reasons to believe that our results are highly similar to those of a large range of ectotherm organisms (de Majo et al., 2019; Hall & Warner, 2020; Kuznetsov et al., 2016; Newell, 1966; Spanoudis et al., 2019; Waqas et al., 2020; Wu et al., 2015). We speculate that the reason for this is that developmental mechanisms are highly conserved. As such, temperature fluctuations, and systematic impacts thereon – like climate change and urban heat islands – should be considered in experimental work determining the effects of temperature and its interactions. Given that development time of multiple other ectotherm organisms has been shown to be affected by fluctuating temperatures, these findings may have implications ranging far beyond those for mosquitoes.

Our novel temperature control system (HIHI) allows for a crucial step, when aiming to include ecological realism in experimental setups. Our system provides an economical means to simulate natural fluctuations under field‐like conditions above ambient temperature and provides a major improvement as compared to currently used systems. A remaining question is whether further steps need be taken when emulating climatic conditions. Fluctuations at different mean temperatures have been described to affect ectotherm organisms non‐linearly (de Majo et al., 2019; Kingsolver et al., 2015; Wu et al., 2015), with species‐specific optimal means (Niederegger et al., 2010). As such there is a need to implement thermal fluctuations in experiments on for instance the effects of heat spikes and more complex fluctuations (Greenspan et al., 2016) on (potentially sex‐specific) mortality and assessing severeness of metabolic effects in other organisms. These alterations could be implemented by simply adapting the temperatures within the code. When doing this, we urge future users to verify the interval using a similar pilot as in section 2.2.1 as passive cooling is dependent on a multitude of variables including, but not limited to, ambient temperature, humidity, and volume/surface ratio. The current setup does not allow for active cooling as such equipment is costly and likely introduces significant additional disturbance. Therefore, without adaptations, the current setup is limited to temperature regimes above ambient temperatures, or experiments using phenological forcing. Regions with distinct seasonal temperature variations may thus be better suited for the proposed equipment in its current form. As such, alterations would be necessary if cooling below ambient temperature is desired. For instance, by circulating of cooling water via heat exchangers or using a jacketed mesocosm (Potter, 2023; Silverberg et al., 1995). Alternatively, ambient heating could be minimized by using shading cloth (Schrama et al., 2018; Sukiato et al., 2019). Adaptation to terrestrial setups might be preferable, which can be done similar to the works of Cheng et al. (2011) and Greenspan et al. (2016), i.e., by introducing a humidity sensor and ultrasonic transducer. Similarly, more complex systems should be considered to assess incorporating interactive effects related to water flow of, e.g., presence of organic matter, salinity, vegetation, etc. As such our tool provides a reliable and cost‐effective means for a broad range of applications. This calls for regular reflection regarding recent technological advances as well as a certain amount of stubbornness from (PhD‐)students to create such innovations.

## AUTHOR CONTRIBUTIONS

**Sam P. Boerlijst:** Conceptualization (equal); data curation (lead); formal analysis (lead); investigation (lead); methodology (lead); project administration (equal); software (lead); validation (equal); visualization (lead); writing – original draft (lead); writing – review and editing (lead). **Eline Boelee:** Funding acquisition (equal); supervision (equal); writing – original draft (equal); writing – review and editing (equal). **Peter M. van Bodegom:** Formal analysis (equal); funding acquisition (equal); supervision (equal); writing – original draft (equal); writing – review and editing (equal). **Maarten Schrama:** Conceptualization (equal); funding acquisition (equal); investigation (supporting); supervision (equal); writing – original draft (equal); writing – review and editing (equal).

## FUNDING INFORMATION

This publication is part of the project “Preparing for vector‐borne virus outbreaks in a changing world: a One Health Approach” (NWA.1160.1S.210) which is (partly) financed by the Dutch Research Council (NWO).

## CONFLICT OF INTEREST STATEMENT

The authors declare that they have no competing interests.

## Supporting information


Appendix S1:

## Data Availability

The data supporting the findings of this study, R‐script, and the operational code are available from the Zenodo repository: https://zenodo.org/doi/10.5281/zenodo.10724529. Build instructions are available within the article its supplementary materials.
