# Supplementary material for: In the heat of the moment: Including realistic thermal fluctuations results in dramatically altered key population parameters
Source: Ecol Evol. 2024 Aug 27;14(8):e70124. doi: 10.1002/ece3.70124 (PMC11349485; doi:10.1002/ece3.70124)
Supplement: Supplementary file 1 — Appendix S1: [file ECE3-14-e70124-s001.docx]

**Supplementary information**

**Section 1** Supplementary figures and tables


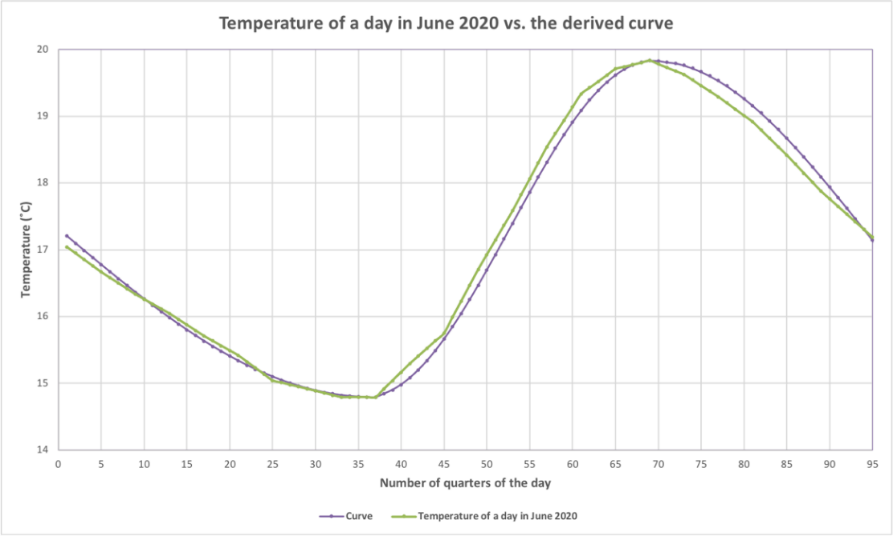


**Figure S1** Average surface water temperature of a day in June 2020 at the experimental site (green). From this data a sinusoidal curve was constructed using the day-length, mean, minimum, maximum temperature, and interval (purple).

**Figure S2** Overview of the experimental design. The circles show the containers with treatments with increasing temperature fluctuation. There was one-meter distance between all containers. The temperature sensors indicate DS18B20 sensors used in real time by the HIHI (for Curve and Curve2) or thermostat (for Constant and Block) to activate/deactivate the heaters per treatment.

**Table S1** Proportion of degree days in each of the treatments during the calibration pilot

|  | **Prior to calibration** | | **After calibration** | |
| --- | --- | --- | --- | --- |
| Treatment | Degree-days | Proportion respective to constant (%) | Degree-days | Proportion respective to constant (%) |
| Constant | 741.77 | 100.0 | 894.05 | 100.0 |
| Curve 1 | 753.58 | 101.6 | 896.03 | 100.2 |
| Curve 2 | 766.45 | 103.3 | 885.20 | 99.0 |

**Table S2** Estimated regression coefficients and standard errors for each of the tests

| Population Parameter | Formula | Treatment | Estimate | Std. Error | t value | Pr(>\|t\|) |
| --- | --- | --- | --- | --- | --- | --- |
| Survival rate | survival_rate ~ Treatment | (Intercept) | 118.4 | 9.486569 | 12.4808025 | 1.16E-09 |
|  |  | Block | 6.6 | 13.416035 | 0.4919486 | 6.29E-01 |
|  |  | Curve | 25.2 | 13.416035 | 1.8783493 | 7.87E-02 |
|  |  | Curve2 | 10.2 | 13.416035 | 0.7602842 | 4.58E-01 |
| Sex ratio | sex ratio ~ Treatment | (Intercept) | 0.97788 | 0.1040257 | 9.40E+00 | 1.12E-07 |
|  |  | Block | 0.16664 | 0.1471145 | 1.13E+00 | 2.75E-01 |
|  |  | Curve | 0.17644 | 0.1471145 | 1.20E+00 | 2.49E-01 |
|  |  | Curve2 | 0.090245 | 1.56E-01 | 0.5783508 | 5.72E-01 |
| Time to pupation | TTP ~ Treatment | (Intercept) | 13.6 | 5.92E-01 | 22.988 | 1.11E-13 |
|  |  | Block | -0.6 | 8.37E-01 | -0.717 | 4.84E-01 |
|  |  | Curve | -1.8 | 8.37E-01 | -2.151 | 4.71E-02 |
|  |  | Curve2 | -2.6 | 0.8367 | -3.11E+00 | 0.00677 |
| Time to emergence | TTE ~Treatment | (Intercept) | 21.666667 | 0.6374553 | 33.989314 | 7.43E-15 |
|  |  | Block | -7.266667 | 0.8063242 | -9.01209 | 3.33E-07 |
|  |  | Curve | -5.066667 | 0.8063242 | -6.283659 | 2.01E-05 |
|  |  | Curve2 | -6.666667 | 0.8063242 | -8.267973 | 9.31E-07 |

**
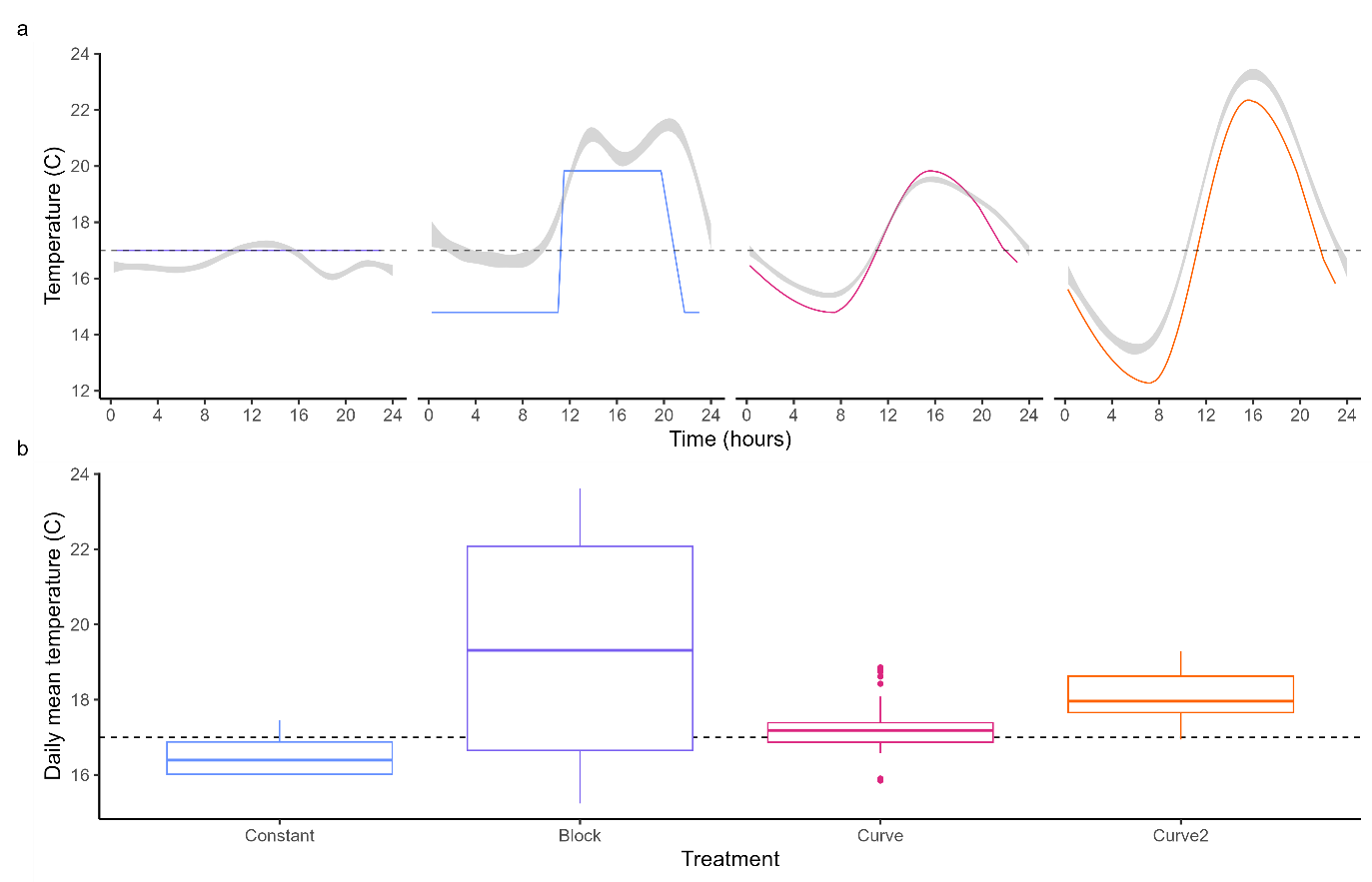
**

**Figure S3** Measured temperatures as compared to the programmed temperatures over time for daily fluctuation regimes of increasing levels of fluctuation for the entire duration of the experiment. Panel a shows the measured temperatures over time with standard error in gray and the programmed regimes in color. Panel b shows the mean daily temperature in color with the standard error (+/- 0.5) for the temperature sensors shown in gray.

**
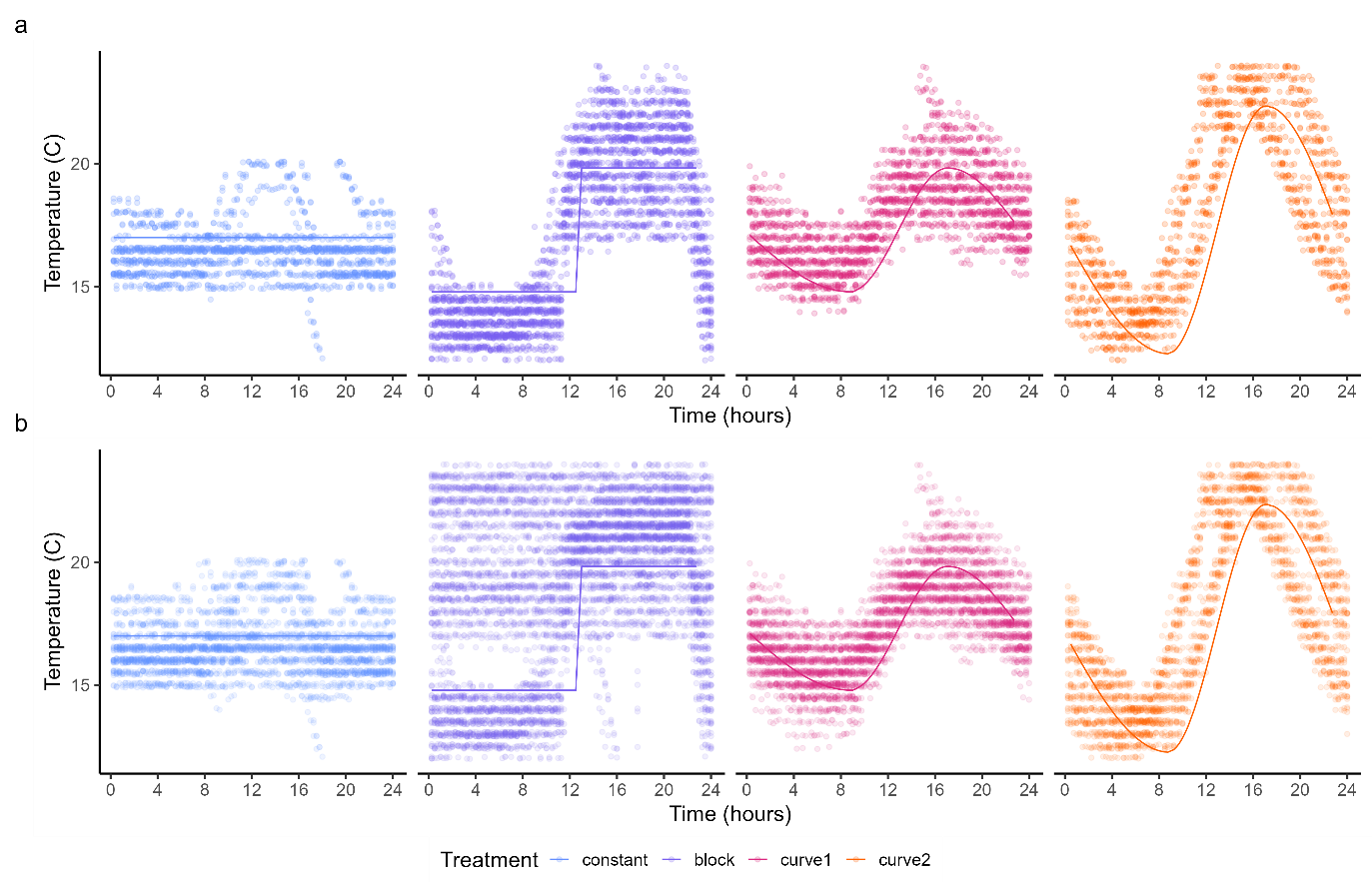
**

**Figure S4** Raw data of measured temperatures as compared to the programmed temperatures over time for the daily fluctuation regimes of increasing levels of fluctuation. Panel a shows the measured temperatures over time and the programmed regimes in as lines up to the short circuit. Panel b shows the measured temperatures over time and the programmed regimes as lines for the entire duration of the experiment.

**
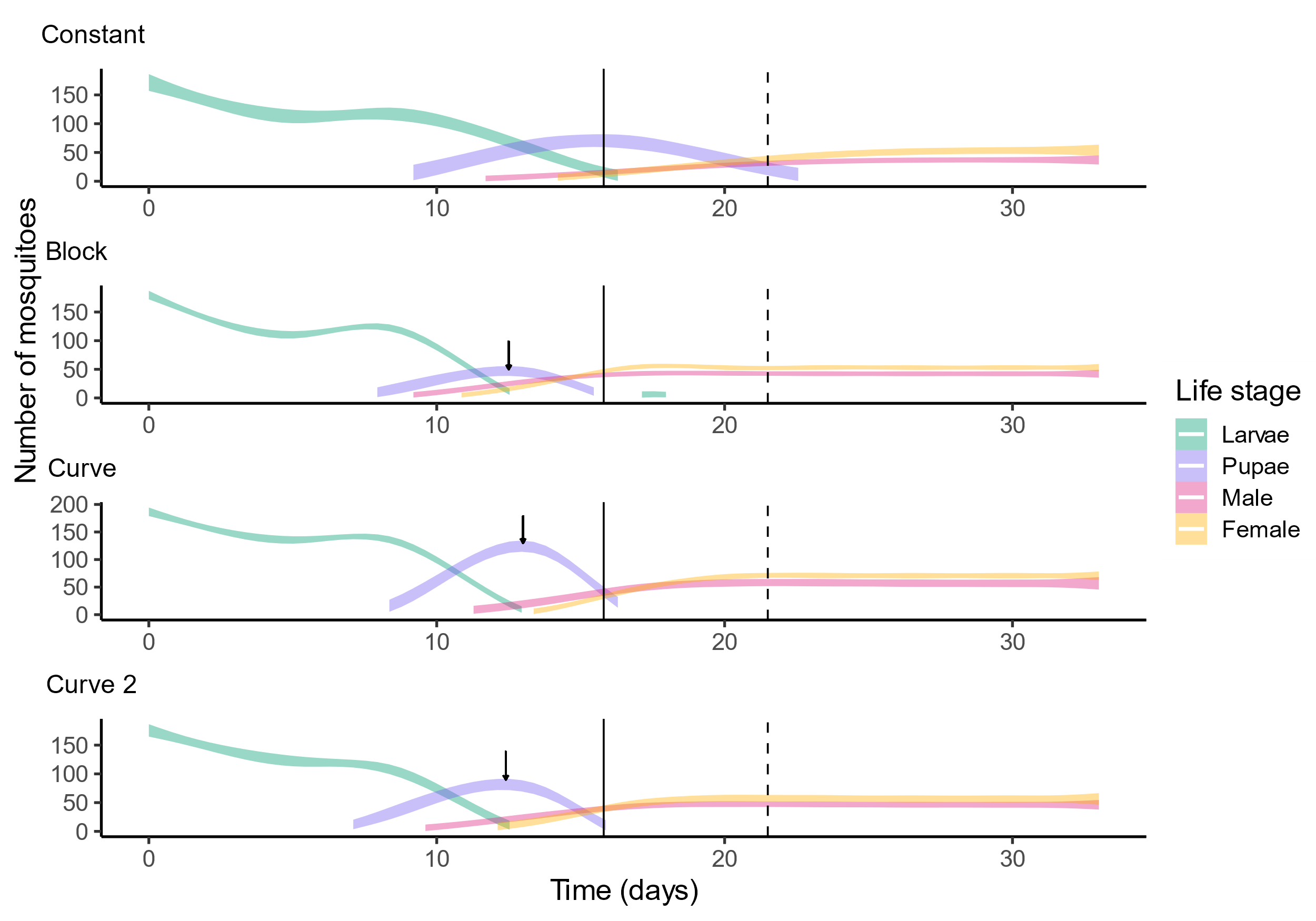
**

**Figure S5** Counts per life stage for constant, block, curve 1 and curve 2. The proportion of the life stages at each timepoint has been extrapolated to the total number of emerged adults for each respective container. As mortality over time was not measured, the sum of the life stages at each timepoint has been assumed to be equal to the total number of emerged adults for each respective container, except for day 0, which is equal to the starting density. Subsequently, for visualization purposes, all mortality is assumed to occur between day 0 and 1. Median time to pupation and 95% pupation in the control treatment are indicated by solid and dashed lines respectively. Median time to pupation in the other treatments are indicated by a red arrow.

**
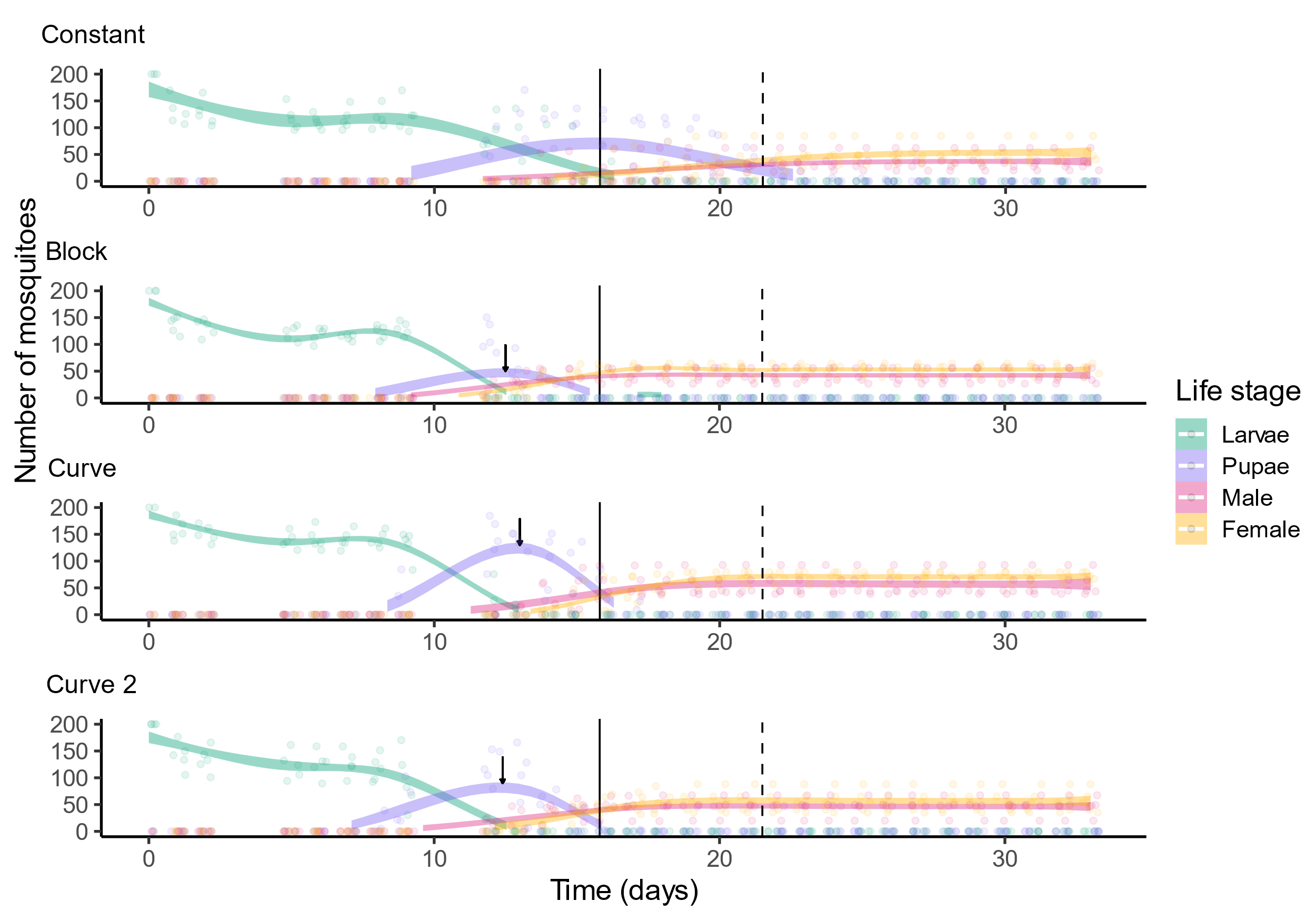
**

**Figure S6** Counts per life stage for constant block, curve 1 and curve 2. As mortality over time was not measured, the sum of the life stages at each timepoint has been assumed to be equal to the total number of emerged adults for each respective container. As mortality over time was not measured, the sum of the life stages at each timepoint has been assumed to be equal to the total number of emerged adults for each respective container, except for day 0, which is equal to the starting density. Subsequently, for visualization purposes, all mortality is assumed to occur between day 0 and 1. Median time to pupation and 95% pupation in the control treatment are indicated by solid and dashed lines respectively. Median time to pupation in the other treatments are indicated by a purple arrow.

**Section 2** Build instructions HIHI

The following are building instructions for the temperature control module. The module consists of two groups of connections. First, the incoming signals, from the temperature sensors, connected to a common ground (black) and 5V (red), and each a separate digital pin (D8-12) for their signal (yellow). Secondly, the outgoing signal, going to the relay board. The relay board is powered by the Vin (red) and ground (black) and is driven by two data lines from digital pin 3 and 4 activating and deactivating the relays. The relay board is connected using female header pins for easy storage. Optionally, the control module can be mounted on the Arduino like a shield using male header pins (Figure S7).


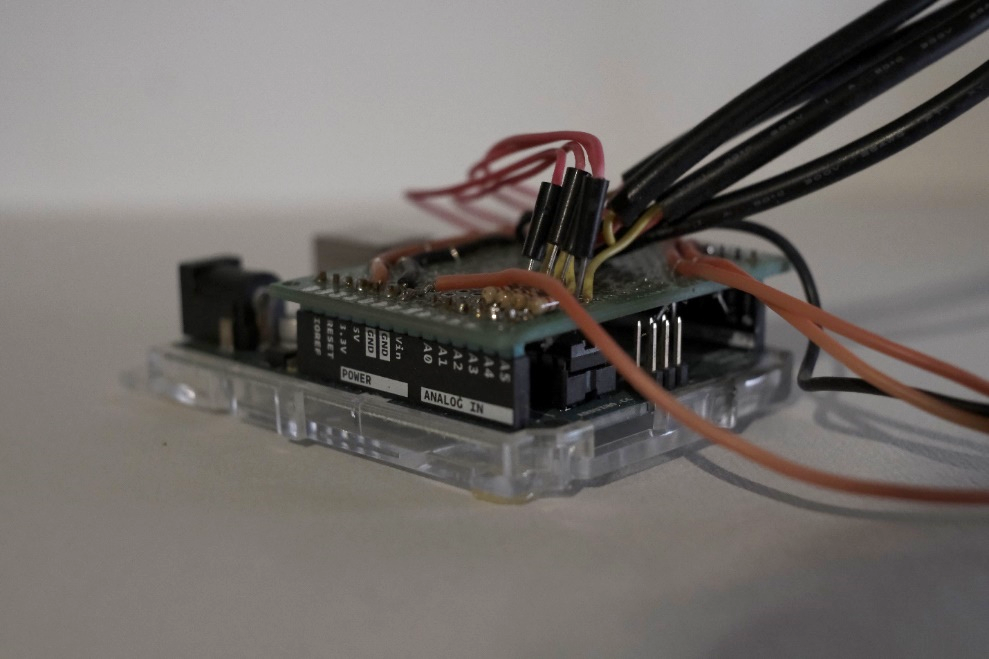


**Figure S7**  Overview of the temperature control module

**Step 1.** Components list

- Arduino uno
- DS18B20 temperature sensor (4x)
- 4 Channel octocoupler relay interface board (e.g. 2ph109375a)
- Resistors 470 Ω (4x)
- perfboard
- Red and black insulated wire (approx. 50cm each)
- Soldering iron and solder
- Female header pin 1x6; as connector to the relay board
- Optional: male header pin (1x14 and 1x8 as shown or 1x1 for each of the four corners; to mount the PCB to the Arduino)
- Optional: 20cm metal wire (for cable management and to secure the wires to the relay module)

**Step 2.** Circuit diagram


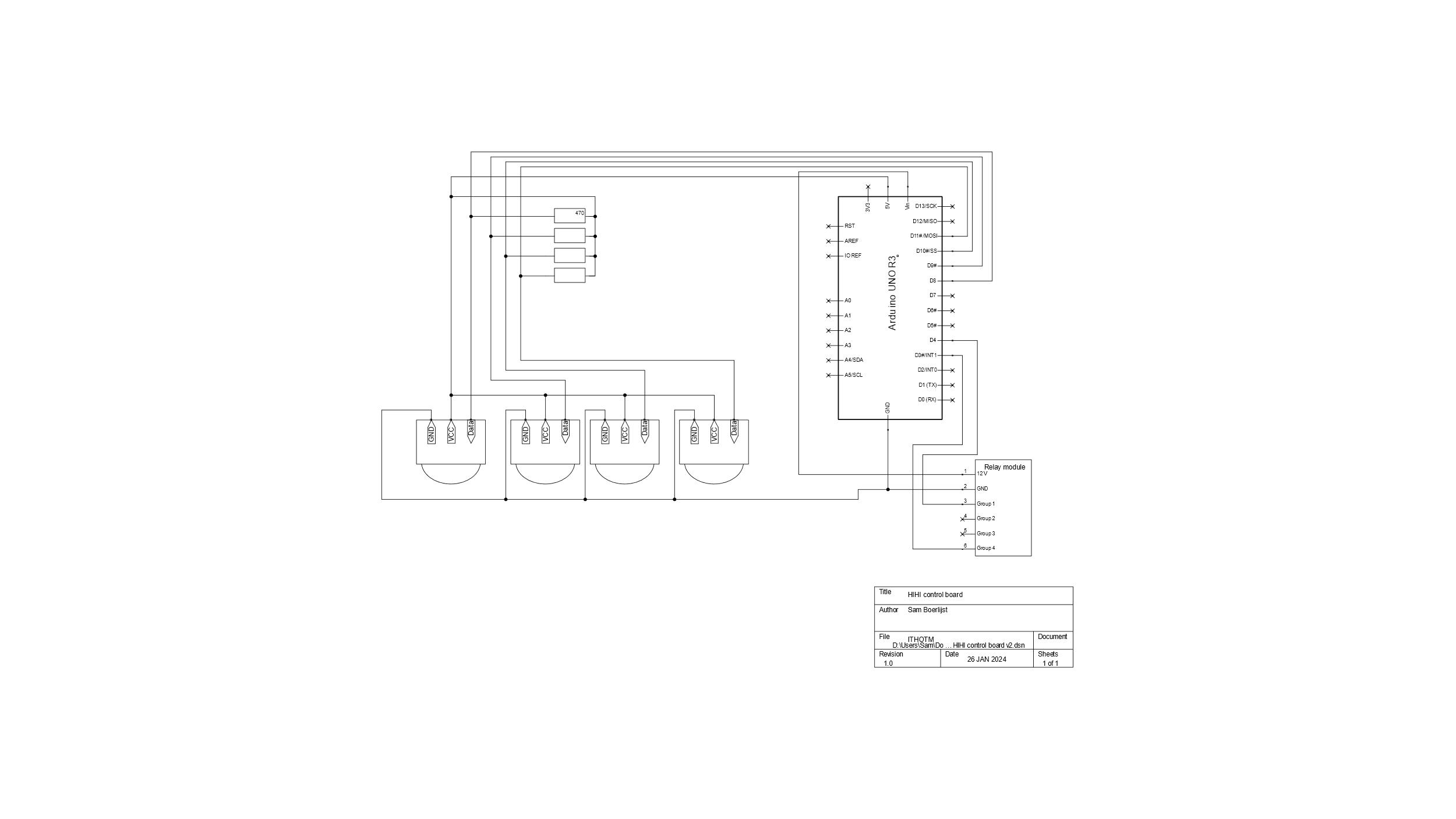


**Figure S8**  Schematic overview of the temperature control module, with a circuit diagram (left) and an example of the corresponding physical layout (right).

**Step 3.** Building process

- Insert all components into the perf board in a similar arrangement to the circuit diagram (Figure S8,9) and bend the legs to prevent them from falling out.
- Optionally, insert the header pins into unused connections on the Arduino. Lay the PCB on top so that it rests flat on the header pins.
- Connect all wires:
  - Relay module: Vin - 12V, GND – GND, D3 – Relay 1, D4 – Relay 2
  - Sensor1: GND – GND, 5V – VCC, D8 & R1 – Data
  - Sensor2: GND – GND, 5V – VCC, D9 & R2 – Data
  - Sensor3: GND – GND, 5V – VCC, D10 & R3 – Data
  - Sensor4: GND – GND, 5V – VCC, D11 & R4 – Data
  - Resistors: 5V – R1 & R2 & R3 & R4
- Solder the connections alternating between components, so that they can cool sufficiently.
- Optionally, cut the metal wire into 4cm strands and secure the wires leading to the relay module at the bottom of the control unit, so that stress on the wires will not damage the solder joint. You may use any remaining wire for cable management.
- Insert the female header pins into the relay module (Figure S10) and solder the respective wires in place. Take care not to heat each connection for too long as this may cause the plastic to melt.


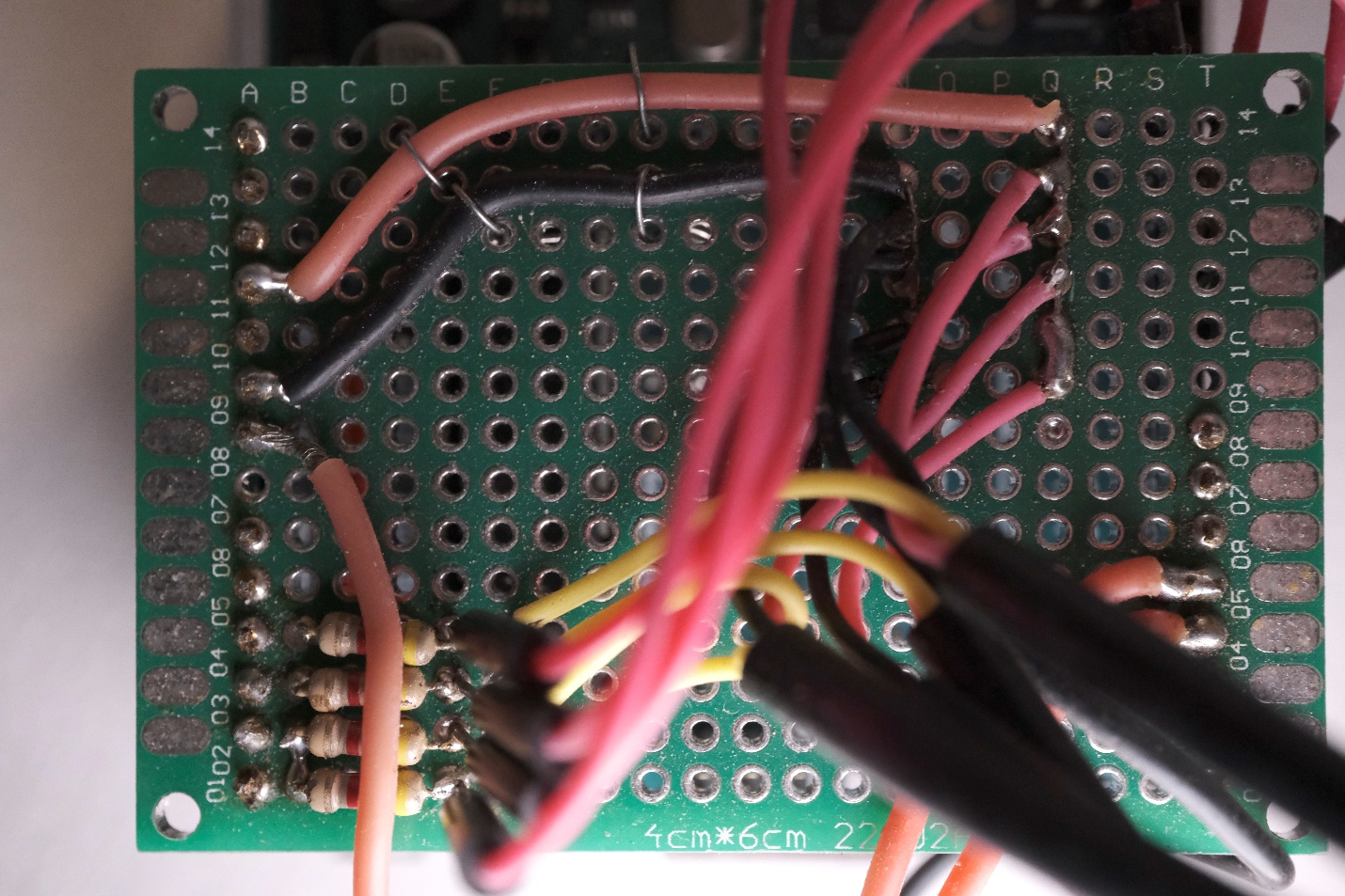


**Figure S9** Overview of the control module.

Connections are indicated using the perf board its indices from A-T (left-right) and 1-14 (bottom-top). The ground (A9) and 5v (A11) connections are split across the temperature sensors at the top (N9-13, Q9-14). Their data lines in yellow at the bottom left (E1-5) are connected directly to digital pins 8-12 at the top right (T11-14), and via resistors at the bottom left (B1-5) to the 5v connector (via the underside of the board; B11). The relay module is powered by the Vin (B8) and ground (N9) and driven by digital pins 3 and 4 of the Arduino (T4,5).

**Step 4** Connect the power strips

- Using a set of pliers, remove 3cm of the outer insulation of each power strip, at roughly 10cm from the plug.
- Cut the blue wire and strip 1cm of insulation of each end.
- Screw open the common and normally closed ports of the respective relays, insert one end of the blue wire into each of the ports and screw the ports shut to secure the wire.


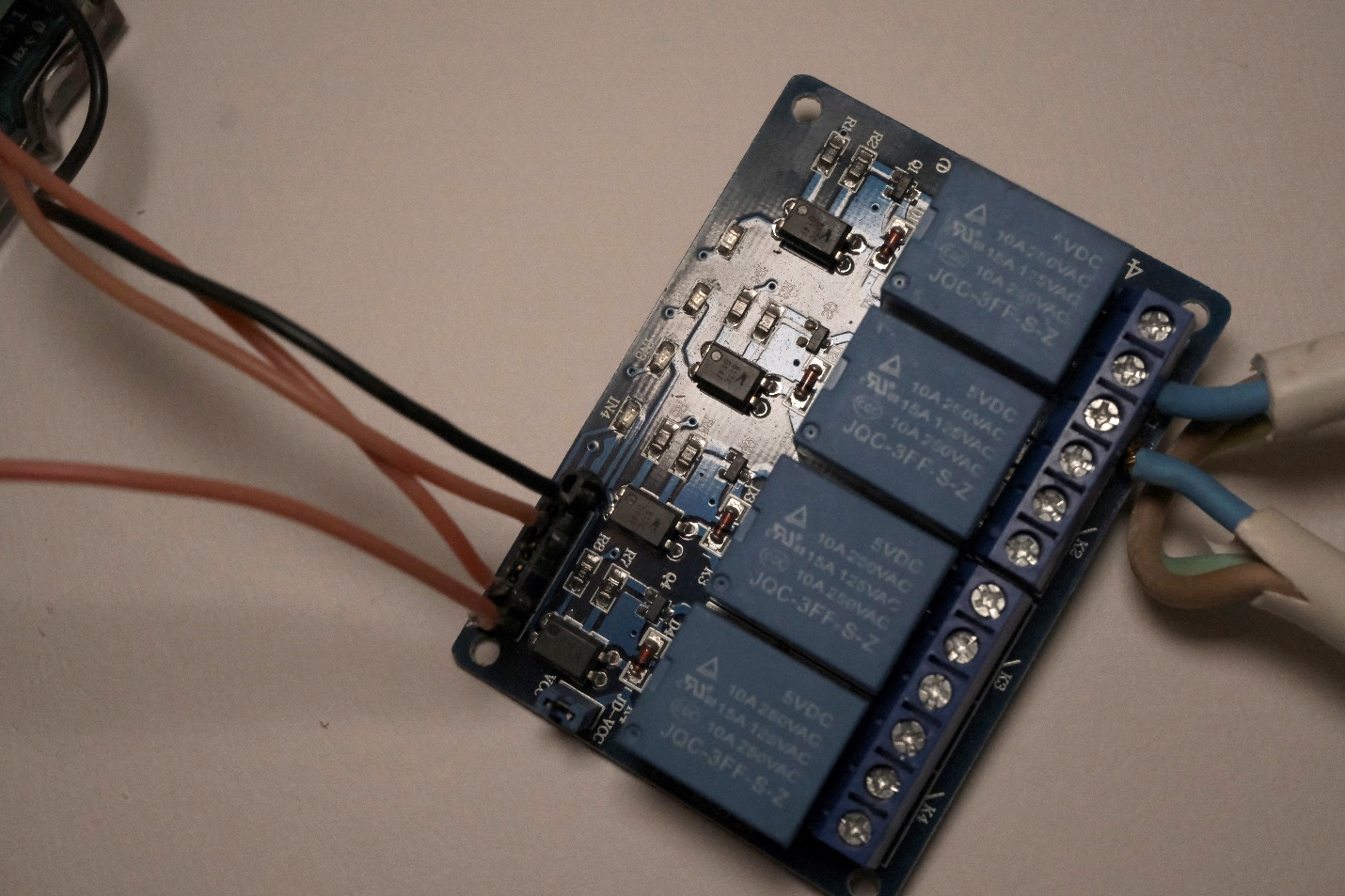


**Figure S10** Overview of the relay module.

The input from the control board (left) consists of (from top to bottom) the ground in black, data line relay 1 in red, data line relay 4 in red and 5v in red. The output to the power strip (right), here shown for relay 1, is connected to the common (middle) and normally closed (NC; bottom) port of the relay.

**Step 5.** Upload the script

Connect the Arduino using the supplied USB connector to your computer and upload the script using Arduino IDE.

**Step 6.** Check functionality

- Prepare a glass of cold water
- Power the Arduino.
- Take the temperature sensors into your hand and heat them up to body temperature.
- Confirm that the relays open by auditory (hearing the relay ‘click’) and visual cues (using the led indicator).
- Drop the sensors into the cold water
- Confirm that the relays close by auditory (hearing the relay ‘click’) and visual cues (using the led indicator).
